# Application of the sliding window method and Mask-RCNN method to nuclear recognition in oral cytology

**DOI:** 10.1186/s13000-022-01245-0

**Published:** 2022-08-02

**Authors:** Eiji Mitate, Kirin Inoue, Retsushi Sato, Youichi Shimomoto, Seigo Ohba, Kinuko Ogata, Tomoya Sakai, Jun Ohno, Ikuo Yamamoto, Izumi Asahina

**Affiliations:** 1 Department of Oral Radiology and Biomedical Informatics, Nagasaki University Graduate School of Biomedical Sciences, 1-7-1, Sakamoto, Nagasaki-City, 852-8501 Japan; 2 Kouguchi Dental Clinic, 1-11-11, Watanabe-Dori, Chou-ku, Fukuoka-City, 810-0004 Japan; 3 Dentistry and Oral Surgery, Hirose Hospital, 1-21-11, Watanabe-Dori, Chou-ku, Fukuoka-City, 810-0004 Japan; 4 Mechanical Engineering Program, Department of Advanced Engineering, Nagasaki University Graduate School of Engineering, 1-14, Bunkyo-machi, Nagasaki City, 852-8521 Japan; 5 Nagasaki University Graduate School of Engineering, 1-14, Bunkyo-machi, Nagasaki City, 852-8521 Japan; 6 Department of Regenerative Oral Surgery, Unit of Translational Medicine, Nagasaki University Graduate School of Biomedical Sciences, 1-7-1, Sakamoto, Nagasaki City, 852-8501 Japan; 7 Research Center for Regenerative Medicine, Fukuoka Dental College, 2-15-1, Tamura, Sawara-ku, Fukuoka City, 814-0193 Japan

**Keywords:** Oral cytology, Artificial intelligence, Sliding window method, Mask-RCNN

## Abstract

**Background:**

We aimed to develop an artificial intelligence (AI)-assisted oral cytology method, similar to cervical cytology. We focused on the detection of cell nuclei because the ratio of cell nuclei to cytoplasm increases with increasing cell malignancy. As an initial step in the development of AI-assisted cytology, we investigated two methods for the automatic detection of cell nuclei in blue-stained cells in cytopreparation images.

**Methods:**

We evaluated the usefulness of the sliding window method (SWM) and mask region-based convolutional neural network (Mask-RCNN) in identifying the cell nuclei in oral cytopreparation images. Thirty cases of liquid-based oral cytology were analyzed. First, we performed the SWM by dividing each image into 96 × 96 pixels. Overall, 591 images with or without blue-stained cell nuclei were prepared as the training data and 197 as the test data (total: 1,576 images). Next, we performed the Mask-RCNN by preparing 130 images of Class II and III lesions and creating mask images showing cell regions based on these images.

**Results:**

Using the SWM method, the highest detection rate for blue-stained cells in the evaluation group was 0.9314. For Mask-RCNN, 37 cell nuclei were identified, and 1 cell nucleus was identified as a non-nucleus after 40 epochs (error rate:0.027).

**Conclusions:**

Mask-RCNN is more accurate than SWM in identifying the cell nuclei. If the blue-stained cell nuclei can be correctly identified automatically, the entire cell morphology can be grasped faster, and the diagnostic performance of cytology can be improved.

## Background

In recent years, remarkable progress has been made in the utilization of artificial intelligence (AI) in medicine. The use of AI for image diagnosis [1] and pathological diagnosis [2] is increasing. The mortality rate of oral cancer remains high, with 15,000 people affected and approximately 7,000 deaths reported per year in Japan [3]. Although the oral cavity can be easily observed and palpated, treatment initiation is often delayed because oral cancer is not well recognized owing to its rarity, and early-stage oral cancer is treated as stomatitis, whose appearance is similar to that of oral cancer.

Oral cytology is a diagnostic technique in which lesions are rubbed in order to collect cells, and the atypicality of the stained cells is assessed, allowing the diagnosis of Class I to V lesions (Papanicolaou classification). Class I and II lesions are usually considered inflammatory reactions, while Class III and above lesions require biopsies to obtain a definitive diagnosis of dysplasia or malignancy. Oral cytology is one of the most useful modalities for the early detection of oral cancer. Although cytology is a simple and reproducible technique, the accurate diagnosis of cellular atypia is based on the experience of a pathologist and may vary among these experts. These factors are barriers to performing cytology among general practitioners. The development of a system using AI to assist in detecting abnormalities in digital cytological images could solve this problem. During Papanicolaou staining for cytological screening of oral cancer, the blue-stained cells were located on the basal side and the red-stained cells on the epidermal side. The detection and recognition of blue-stained cells is required for the early detection of dysplastic changes. As the atypia of the cells increased, the nucleus-to-cytoplasm (N/C) ratio increased. In addition, a significant variation was observed in the cytoplasmic staining, while only a slight variation was observed in the nuclear staining. Therefore, it is necessary to identify the nuclei of blue-stained cells in cytopreparation images. Since Class III–V lesions should be considered for biopsy, Class I and II lesions should be distinguished from Class III and V lesions. This study aimed to detect the nuclei in blue-stained cells and to identify their classification.

During image recognition using AI, the objects are detected and identified. Two methods are used for proposing the object region candidates: the sliding window method (SWM) [4–10] and the mask region-based convolutional neural network (Mask-RCNN) [11]. The SWM extracts candidate regions by shifting the region to a fixed size at a fixed pixel interval. The extracted regions are applied to an image discriminator to determine the presence of important objects in the window. This is a slow but reliable method. Mask-RCNN(Mask Region-based Convolutional Neural Network) is a method used for object detection and pixel-by-pixel segmentation of images, and a study detailing this method was selected as the best study at the 16th International Conference on Computer Vision [10] Mask-RCNN can detect object-like regions in an image and their classes. Object-like regions were detected in large numbers; they were obtained by dividing the image into specific regions and evaluating them thoroughly. By narrowing down the image to those regions where the “nucleusiness” is higher than the threshold value or those regions with the highest “cell nucleusiness” from a group of regions where the overlap between regions is higher than the threshold value, highly accurate results can be obtained. Therefore, we aimed to identify the possibility of classifying the presence or absence of cell nuclei using Mask-RCNN. In this study, we compared the performance of SWM and Mask-RCNN in detecting cell nuclei in oral cytological images.

## Methods

### Dataset preparation

Specimens for oral cytology of oral mucosal diseases were collected at the Department of Oral and Maxillofacial Surgery, Nagasaki University Hospital. Abrasion (conventional) cytology and liquid cytology were performed simultaneously at the time of specimen collection. For conventional cytology, only a small number of cells were collected, and a significant overlapping of cells was observed. Many foreign bodies including debris were also detected. However, a large number of cells were retrieved, and the debris was removed from the samples for liquid cytology, which made it easier to identify the cells. In this study, liquid cytology was performed. The microscopic images of cytopreparation were taken using a Nikon Eclipse Ti-S inverted microscope with a DS-Ri1 digital camera (Nikon Corp. Tokyo, Japan) equipped with a 40 × objective lens. The images were saved in tagged image file format at 1024 × 1280 pixels.

### Papanicolaou classification of oral cytology [11]

An oral pathologist evaluated all images. The Papanicolaou classification system comprises five classes. Class 1 tumors do not contain abnormal or atypical cells. Class 2 tumors have atypical cells, but are not malignant. Class 3 tumors are suspected to be malignant but cannot be ruled out. Class 4 tumors are highly suspicious for malignancy. Class 5 tumors are almost certainly malignant. In addition, evaluation is made based on cytoplasmic staining, cytoplasmic luminosity, cytoplasmic thickness and structure, cell shape, cell size difference, N/C ratio, nuclear shape, nuclear size difference, nuclear limbus appearance, nuclear number, chromatin amount, distribution and pattern, and nucleolus appearance.

### Application of SWM

**Fig. 1:**
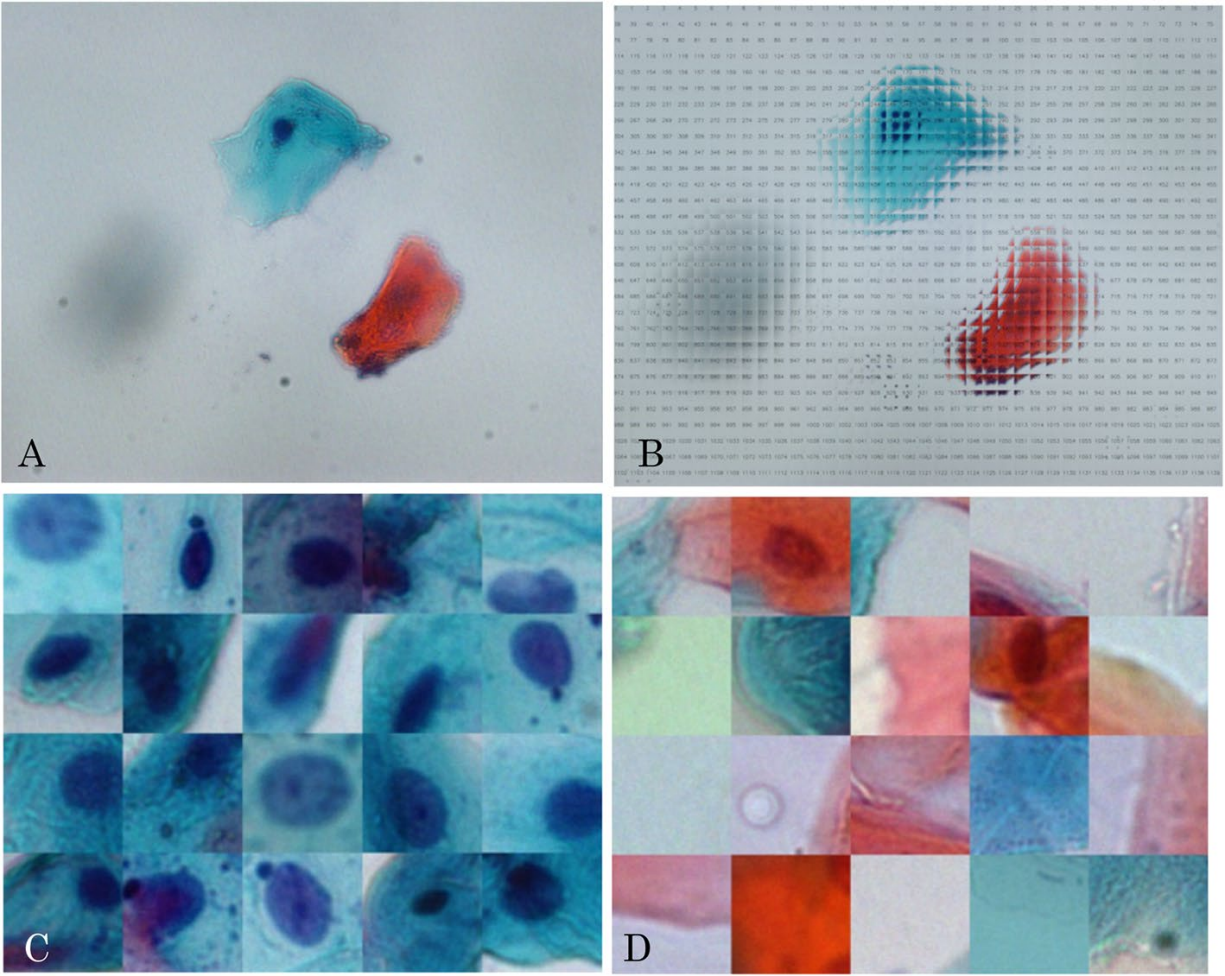
Dataset preparation for the sliding window method (SWM). **A** Original image of liquid-based oral cytology (1280 × 1024 pixels). **B** SWM applied. The size of the sliding window is 96 × 96 pixels. One window contains the cell nucleus, while another window does not. **C** Images of windows containing only the cell nuclei. **D** Example of a window without a cell nucleus

First, an oral cytology image was divided into 96 pixels (Fig. 1A and B). The images were manually sorted into those that contained cell nuclei and those that did not, and a dataset for classification was created using a convolutional neural network (CNN). Only images containing more than half of the cell nuclei were extracted to determine whether they were cell nuclei. During cytological examination (Papanicolaou staining), only the blue-lineage cell nuclei were extracted, which is important for the early detection of dysmorphic cells (basal cells).

Therefore, we only extracted the nuclei of the blue-stained cells. A total of 788 images with (Fig. 1C) and without (Fig. 1D) nuclei were prepared. The data of cells with and without cell nuclei were then randomly divided into training and test data. Overall, 591 images of cells with or without nuclei were prepared for the training group, while 197 images were prepared for the evaluation group (1,576 images in total).

**Fig. 2:**
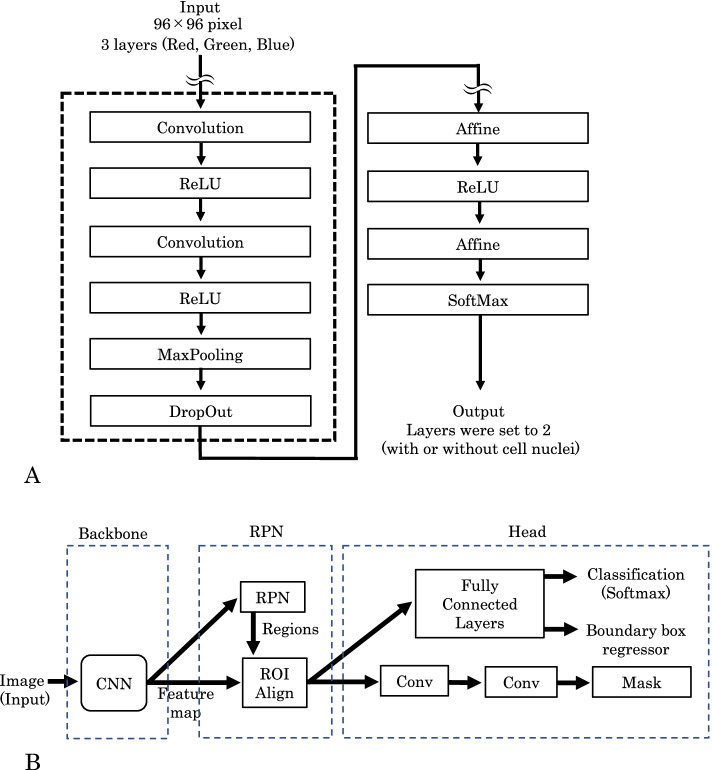
Constructed image classifier. **A** Image classifier using the sliding window method. Categorical cross-entropy was used as the loss function, and ADAM software was used to update the parameters. The input layer was set to 96 × 96 with three channels (red, green, and blue), while the output layer was set to two, one containing the cell nuclei and the other without the cell nuclei, respectively. **B** Image classifier with mask region-based convolutional neural network method (Mask-RCNN). Mask-RCNN consists of three regions: backbone, region proposal network (RPN), and head. The backbone extracts features of the input image. The RPN determines whether each fixed region and the overlap of the regions are correct. The head layer pools the candidate RPN regions to the same image size and then calculates the probability for each class of cytology. CNN, convolutional neural network; ROI, region of interest; Conv, convolutional layer

Second, we constructed an image classifier, the CNN (Fig. 2A). The input image was set to 96 × 96 pixels with three channels (red, green, and blue), while the output layer was set to two channels (with or without cell nuclei). Categorical cross-entropy was used as a loss function to calculate the label and output errors, and optimization was performed using the ADAM software to update the network parameters [12, 13]. To obtain more accurate loss function values and results, we increased the number of epochs and attempted to drop out of the system to avoid overlearning. We tried to deepen the network (convolution, pooling, and increasing the set of activation functions) and varied the activation functions accordingly.

### Mask-RCNN method application

To train the Mask-RCNN, mask images were required in the teacher images. As the purpose of this study was to distinguish Class II cells from Class III cells, a dataset of 130 images (original and mask images) of each class was constructed.

The structure of the image identifier with Mask-RCNN is shown in Fig. 2B. Mask-RCNN was largely divided into three layers: backbone, region proposal network (RPN), and head. The backbone extracts features of the input image. The RPN determines whether each fixed region is correct and whether the overlap of regions is correct. The head layer pools the candidate RPN regions to the same image size and then calculates the probability for each class.

### Diagnostic performance of the AI system

The AI systems were implemented on an NVIDIA GeForce RTX 2080Ti (NVIDIA Corp., Santa Clara, CA, USA) with an Intel Core i9-9900 K processor (Intel Corp., Santa Clara, CA, USA) and 64 GB of memory.

## Results

### Results of SWM

The CNN training results are shown in Table 1. The CNN with the highest proportion of correct answers in the training data was No. 6 (99.8%); however, the accuracy of the test data was 89.1%, which was less accurate. This finding is due to overlearning. The CNN with the highest accuracy, without overlearning, was No. 2, which increased the proportion of correct answers to approximately 93% of the test data.

**Table 1:** Results of the sliding window method (SWM)

No	Activation function	Epoch	No of layers	Dropout	Value of the training loss function	Percentage of correct answers for the training group	Value of the loss function of the test group	Percentage of correct answers in the test group
1	ReLU	5	18	0.25	0.3356	0.8668	0.3266	0.8654
2	ReLU	20	18	0.25	0.0891	0.9562	0.2104	0.9314
3	ReLU	50	18	0.25	0.0526	0.9851	0.6082	0.9111
4	ReLU	5	24	0.25	0.351	0.8524	0.3188	0.8705
5	ReLU	20	24	0.25	0.0798	0.9251	0.1958	0.9314
6	ReLU	50	24	0.25	0.0628	0.9982	0.4614	0.8908
7	ReLU	5	18	0.5	0.3752	0.8226	0.3225	0.8705
8	ReLU	20	18	0.5	0.2151	0.9198	0.2514	0.9213
9	Sigmoid	20	18	0.25	0.7456	0.4659	0.7285	0.4785

The results obtained using the SWM and convolutional neural network (CNN) methods are shown. To improve the accuracy of the loss function values and correctness of the answer rate, several dropouts were attempted to increase the number of epochs and avoid overlearning. We also deepened the network by increasing the number of convolution, pooling, and activation functions

The CNN with the highest number of correct answers in the training data was No.6 (99.8%), whereas the percentage of correct answers in the test data was 89.1%. This is thought to be due to overlearning. The CNN with the highest accuracy and without overlearning was No. 2 (93.1% correct answers on the test data)

**Fig. 3:**
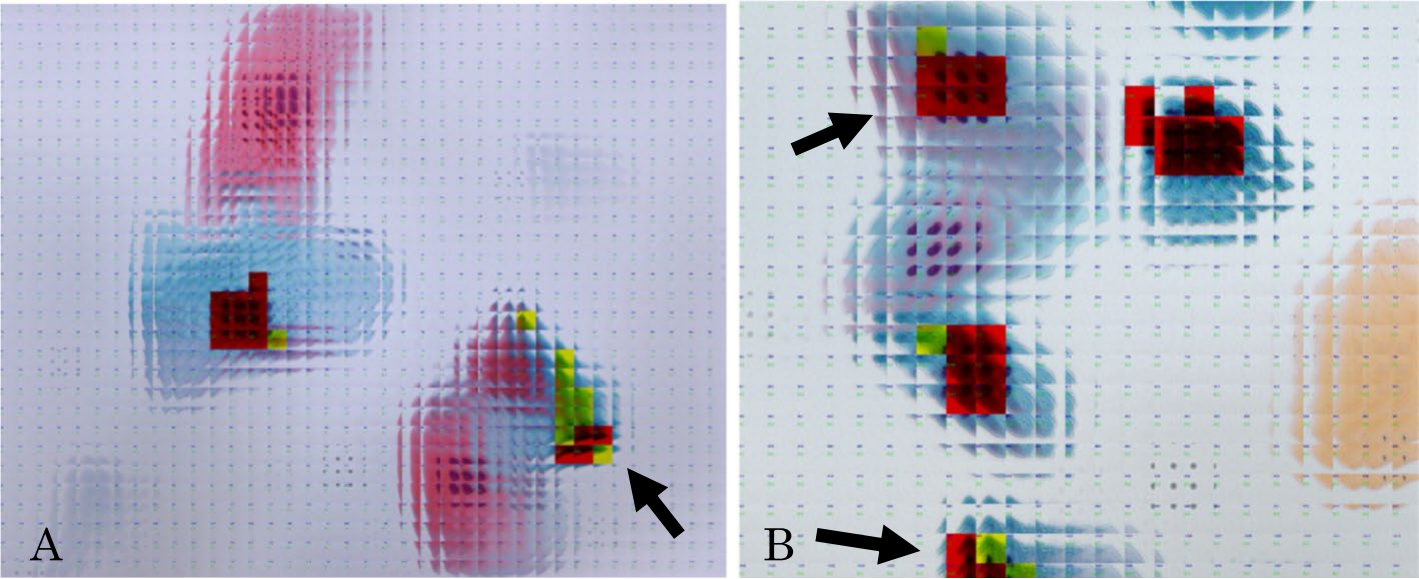
Example of images in which the convolutional neural network was identified as the cell nuclei. Red indicates > 90% probability of being a cell nucleus, while yellow indicates > 50% probability of being a cell nucleus. **A** An example of detection from background (arrow). **B** An example where a nucleus was not detected (arrow)

Figure 3A and B shows colored images indicating the CNN as cell nuclei. Red indicates that the probability of being a cell nucleus is more than 90%, while yellow indicates that the probability is more than 50%. As shown in Fig. 3A, this finding was not sufficiently accurate because it reacted to the background image and did not react to the blue cell nuclei, as shown in Fig. 3B. We also considered the possibility of overlearning owing to the presence of multiple cell nuclei in a single image. As a countermeasure to these drawbacks, we used Mask-RCNN.

### Results of Mask-RCNN

**Fig. 4:**
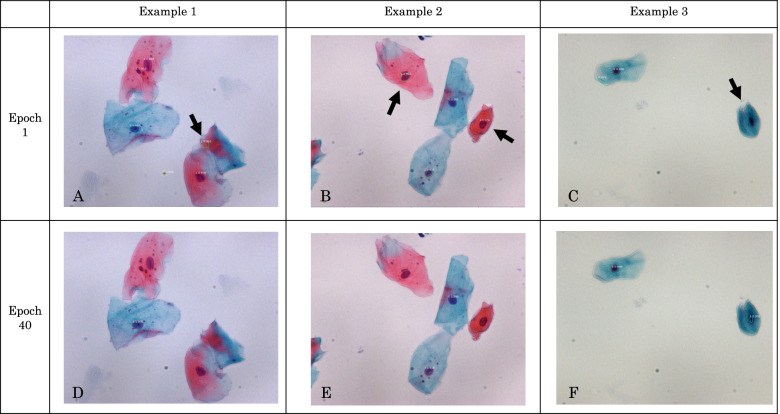
Detection of cell nuclei using the region-based convolutional neural network method. Epoch 1 indicates the start of learning, while epoch 40 indicates the end of learning 40 times. Epoch 1 can identify the parts other than the cell nuclei (**A**), red cell nuclei (**B**), and blue absent cell nuclei (**C**). Learning allowed the discrimination of the blue-tinted nuclei alone (**D**, **E**, **F**), and the discrimination performance was improved

At the beginning of the course (epoch 1), the loss was 0.8904, and the validation loss was 0.7262. At the end of the course (epoch 40), the loss was 0.4547, and the validation loss was 0.4820. The loss function was lower at the end (40 epochs) compared with that at the beginning (one epoch). When the prepared images were examined using the obtained model, we succeeded in detecting the cell nuclei, as shown in Fig. 4. In the other images, the accuracy of this method was higher than that of the SWM model (Fig. 2A). This result shows that Mask-RCNN can be applied to the detection of Papanicolaou-stained cell nuclei in the oral cytology of liquid-based samples.

## Discussion

### CNN

The basic structure of the CNN used includes convolutional, pooling, dropout, and affine layers [12]. The generalization capability was improved so that it was possible to detect the features in other images as well. After applying these convolutional and pooling layers, the affine layer performed the compilation (all joins), while the softmax function was used to output the results. The more convolutional and pooling layers are used, the higher the expected accuracy; however, gradient loss occurs during training, which deteriorates the accuracy. Because the error backpropagation method is used to train the neural network, the gradient is lost, learning is not possible, and the accuracy is not improved. The dropout layer prevents overlearning. Overlearning of the training data can be avoided by deliberately setting the output data to zero during the layer-to-layer transfer. Because the output data that dropped out changed randomly as the training was repeated, a new one was learned each time, and overlearning of the training data was avoided. Considering the above finding, a CNN was constructed, as shown in the figure.

### Comparison of the results

**Fig. 5:**
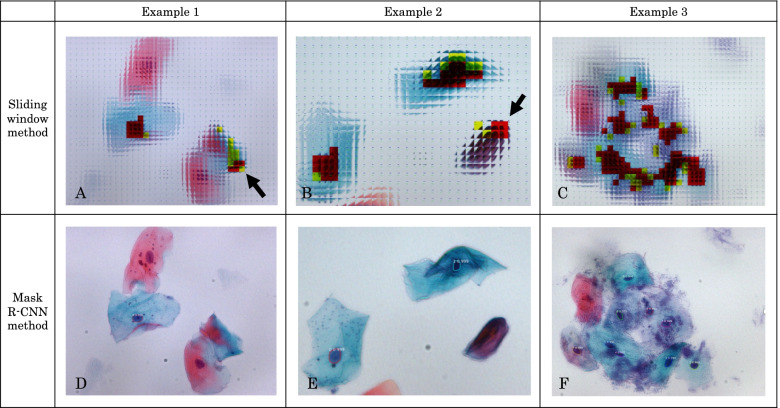
Comparison of the detection accuracy between the sliding window method (SWM) and mask region-based convolutional neural network (Mask-RCNN) method using the same image. **A**, **B**, and **C** show the SWM, while **D**, **E**, and **F** show the Mask-RCNN (at the end of epoch 40). **A**, **B** The non-nuclear regions (arrows) are indicated in red, while **C** discriminates the nucleus regions in red but in a wider range. By contrast, **D**, **E**, and **F** are only identified as cell nuclei in blue

In the detection of cell nuclei, the SWM and Mask-RCNN methods have different criteria for the loss function; therefore, the results of the loss function cannot be compared. Hence, a visual comparison was performed. We randomly prepared 10 whole images of Class II and III tumors and assessed them using the SWM and Mask-RCNN methods based on the following criteria: (1) number of detections, (2) percentage incorrectly detected from backgrounds, (3) average number of undetectable cell nuclear regions per cell nucleus, and (4) average number of regions detected for each cell nucleus (Table 2). The Mask-RCNN method showed lower percentage of background detection and lower average number of undetected regions per cell nucleus. These results suggest that the Mask-RCNN method is more accurate because it can detect the cell nuclei and does not react to extra regions. The average number of detected regions for each cell nucleus in the Mask-RCNN method was one (36/36). This finding indicates that there was no overlap in the number of detected cell nuclei, thus indicating that the data are easy to use for the next stage of training. Figure 5 shows three examples of the visually assessed images. The Mask-RCNN method can detect the outline of a cell nucleus. In other words, the regions other than the cell nuclei were omitted. This makes it easier to extract the features of the cell nucleus only when the training is performed in the next stage. Therefore, the Mask-RCNN method appeared to be more accurate than the SWM in detecting the cell nuclei, and could be applied to the next stage of training. However, the cell nuclei were more accurately detected using the Mask-RCNN method because the Class II and III images used in this study were relatively clean, with little debris for the dataset. The results in cases with large amounts of debris or overlapping cells still need to be determined. If the cell nuclei can be automatically detected, the accuracy of cytological diagnosis can be improved by detecting the cells using an attention mechanism [14].

**Table 2:** Comparison of the results of the sliding window method (SWM) and mask region-based convolutional neural network (Mask-RCNN) method

	Sliding window	Mask R-CNN
Number of detections	320	37
Percentage of incorrectly detected from backgrounds	0.3031 (97/320)	0.027 (1/37)
Average number of undetectable cell nuclear regions per cell nucleus	1.8611 (67/36)	0 (0/36)
Average number of regions detected for each cell nucleus	6.1944 (223/36)	1 (36/36)

Ten images were randomly selected from Classes II and III. Mask-RCNN showed lower background detection rate and lower average number of undetected regions per cell nucleus compared with SWM. The average number of detected regions for each cell nucleus in Mask-RCNN was 1. This indicates that the detected cell nuclei were not duplicated

Diagnosis through cytological examination is based on the cell morphology and the N/C ratio. However, in the present study, the positivity rate was relatively high despite focusing only on the recognition of cell nuclei. This clarified the possibility of classifying cytological diagnoses by focusing on the morphology of cell nuclei.

In the medical field, AI has been investigated for its potential use in diagnostic imaging and pathology. AI may provide more accurate results than the manual reading of CT and chest X-ray images by radiologists [15]. The cytology of cervical cancer has been well studied [2] the number of cervical cancer cases is higher than that of oral cancer cases, making it easier to construct datasets. On the contrary, oral cancer is rare (less than 6 people per 100,000 population in Japan), making it difficult to collect cases and build a dataset. The present study attempted to apply Mask-RCNN to the cytological screening of rare cancers such as oral cancer. If the results of this study can be applied to other rare types of cancer, they will contribute to the early detection of cancer through cytological screening.

## Conclusions

The Mask-RCNN method showed a higher positive detection rate than the SWM for cell recognition in liquid cytology. Moreover, classification could be performed by identifying the cell nuclei.

## Abbreviations

AI: Artificial intelligence
Mask-RCNN: Mask region-based convolutional neural network
N/C ratio: Nucleus-to-cytoplasm ratio
RPN: Region proposal network
SWM: Sliding window method
